# Learning in one minute: survey of the One Minute Wonder Network

**DOI:** 10.1007/s00063-021-00892-3

**Published:** 2022-01-04

**Authors:** Lars Krüger, Thomas Mannebach, Marianne Rahner, Fabian Timpe, Franziska Wefer, Peter Nydahl

**Affiliations:** 1 Continuing Education Intensive Care, Surgical Intensive Care Unit E 0.1, Heart and Diabetes Center NRW, University Hospital of the Ruhr University Bochum, Georgstraße 11, 32345 Bad Oeynhausen, Germany; 2 Surgical Intensive Care Unit E 0.1, Heart and Diabetes Center NRW, University Hospital of the Ruhr University Bochum, Georgstraße 11, 32345 Bad Oeynhausen, Germany; 3 Institute for Public Health and Nursing Research, University Bremen, Grazer Straße 4, 28359 Bremen, Germany; 4 Care Development, Care Directorate, St. Bernward Hospital GmbH Hildesheim, Treibestraße 9, 31134 Hildesheim, Germany; 5 Care Development, Care Directorate, Heart and Diabetes Center NRW, University Hospital of the Ruhr University Bochum, Georgstraße 11, 32345 Bad Oeynhausen, Germany; 6 Nursing Research, Department of Anaesthesia and Operative Intensive Care Medicine, University Hospital Schleswig-Holstein, House V40, Brunswiker Str. 10, 24105 Kiel, Germany

**Keywords:** Evaluation, Continuing education, Intensive care unit, Knowledge transfer, Nursing, Evaluation, Fortbildung, Intensivstation, Wissenstransfer, Pflege

## Abstract

**Background:**

Continuous education of clinicians improves quality of care. One Minute Wonder (OMW) summarize best practice knowledge on one page that can be hung on a wall and can be read during waiting times of just one minute. OMW are a fast, efficient and easy-to-adapt educational method and can easily be shared. Since 2018, an interprofessional network has been set up for OMW in German-speaking countries, but the benefits have not been evaluated yet.

**Aim:**

The primary objective of this evaluation study was to examine whether and to what extent the members of the OMW network used OMW for training in different settings. Secondary objectives were subjective educational gain, OMW as a training method, and OMW-related structures and processes.

**Methods:**

An online survey within the OMW network with 301 members over a period of 3 weeks in 2020 was conducted. Descriptive statistics were used for data analysis.

**Results:**

Response rate was 62.8% (*n* = 191). Most participants have used OMW for < 6 months (32.5%, *n* = 62), developed 1–10 OMW (42.4%, *n* = 81) by themselves and changed them infrequently (43.5%, *n* = 74). Topics were most often nursing interventions (79.6%, *n* = 152), diseases (71.2%, *n* = 136), drugs (64.4%, *n* = 123) and others. Participants reported that OMW extended professional knowledge, stimulated them to reflect on their work and are useful for sharing best practice knowledge. Authors of OMW were most often nurses (53.9%, *n* = 103), who were inspired by the OMW network or by questions of the team.

**Conclusion:**

Participants use OMW in practice to share best practice knowledge.

**Supplementary Information:**

The online version of this article (10.1007/s00063-021-00892-3) includes Table E1.

## Abstract (de)

**Hintergrund:**

Kontinuierliche Fortbildung von medizinischem Personal verbessert die Versorgungsqualität. One Minute Wonder (OMW) fassen praxisbasiertes Wissen auf einer Seite zusammen, hängen an der Wand und können während Wartezeiten in nur einer Minute gelesen werden. Sie sind eine schnelle, effiziente und leicht anzupassende Bildungsmethode und können leicht weitergegeben werden. Seit 2018 wurde für OMW im deutschsprachigen Raum ein interprofessionelles Netzwerk aufgebaut, dessen Nutzen jedoch noch nicht evaluiert wurde.

**Ziel:**

Das primäre Ziel dieser Evaluationsstudie war zu untersuchen, ob und inwieweit die Mitglieder des OMW-Netzwerks OMW für die Fortbildung in unterschiedlichen Settings nutzen. Sekundäre Ziele waren: subjektiv bewerteter Bildungserfolg, OMW als Bildungsmethode sowie OMW-bezogene Strukturen und Prozesse.

**Methode:**

Es wurde eine Onlinebefragung des OMW-Netzwerks mit 301 Teilnehmenden über einen Zeitraum von 3 Wochen im Jahr 2020 durchgeführt. Zur Datenanalyse kamen Methoden deskriptiver Statistiken zum Einsatz.

**Ergebnisse:**

Die Rücklaufquote betrug 62,8 % (*n* = 191). Die meisten Teilnehmenden setzten OMW < 6 Monate ein (32,5 %, *n* = 62), haben 1–10 OMW (42,4 %, *n* = 81) selbst entwickelt und diese selten gewechselt (43,5 %, *n* = 74). Themen waren am häufigsten Pflegeinterventionen (79,6 %, *n* = 152), Krankheiten (71,2 %, *n* = 136), Medikamente (64,4 %, *n* = 123) und andere. Die Teilnehmenden berichteten, dass OMW ihr Fachwissen erweitert, sie zum Nachdenken über ihre Arbeit anregen und für den Austausch von best verfügbarem Wissen nützlich sind. Autoren von OMW waren am häufigsten Pflegende (53,9 %, *n* = 103), die sich vom OMW-Netzwerk oder von Fragen des Teams inspirieren ließen.

**Diskussion:**

Die Teilnehmenden nutzen OMW zum Austausch von bestem praxisbasiertem Wissen.

## Introduction

Continuing education is an important aspect for providing best care by healthcare professionals in intensive care units (ICU). One feasible educational method is the One Minute Wonder (OMW). OMW present healthcare-related information, are hung up in clinicians’ waiting areas, printed on a single page and can be read within a minute.

Since 2018, an interprofessional network has been set up for the development and distribution of OMW in German-speaking countries, but its benefits had not been evaluated yet.

The following study presents the results of the survey of the OMW network and the effects of OMW in different settings—especially the ICU.

### Background

Continuous education of clinicians is an important aspect of professionalism in healthcare, especially in ICUs [21, 27]. One goal is the transfer of knowledge into practice [9]. There are different ways of learning and further training for healthcare workers like e‑learning [18], conferences [25], seminars, workshops or degree courses [2]. Furthermore, short offers from seconds to minutes like SMS [5, 24], videos, or podcasts are also possible [7]. It is not always necessary to be a part of a training course because work-based learning (WBL) also takes place day by day, e.g., during a discussion of a few minutes with colleagues [1] or on the regular work like handovers of nurses or other healthcare professionals [4].

**Fig. 1:**
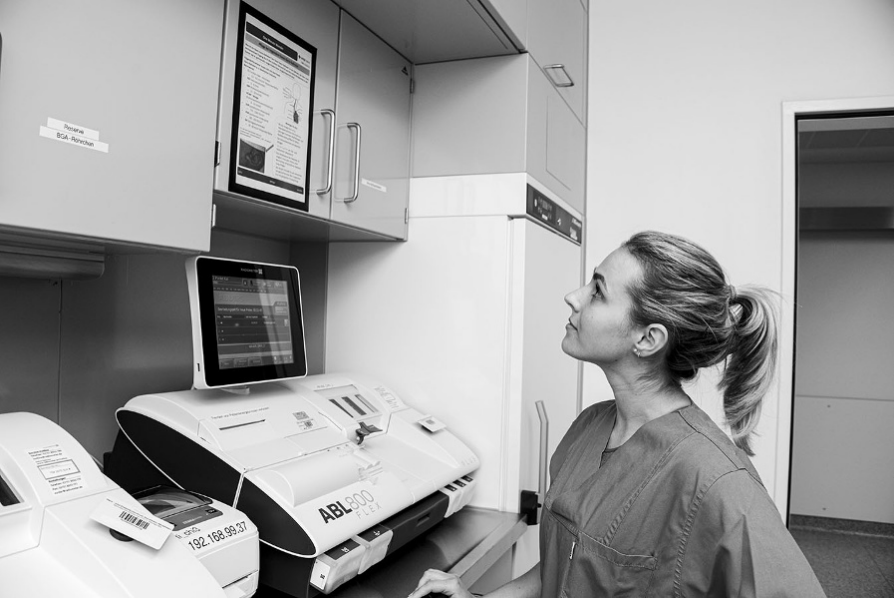
One Minute Wonder (OMW) in an intensive care unit near a blood gas analyzer

One Minute Wonder (OMW) is a training method for the continuous education of clinicians in different healthcare settings, with a focus on critical care [23]. Usually, OMW summarize best practice knowledge such as ventilator settings, blood sugar management, new rules of bandages/dressing changes etc., on one page. OMW include text, tables or figures. OMW are hung up in places with regularly recurring waiting times in daily nursing practice ([23]; Fig. 1). In nursing departments, recurring short waiting times occur in different frequency and duration at certain locations, e.g., at a microwave oven or a laboratory device. OMW are changed in regular intervals of, for example, two weeks. The content of an OMW should be designed to be taken in within about one minute [23]. In addition, the method can be used in many other areas of healthcare, such as the ambulance service [8, 23], gastroenterology [20] or neonatal intensive care units [12]. So-called “OMW managers” can be appointed for coordination and implementation of this educational method [16]. In first pilot projects in anesthesia, OMW were perceived as helpful by caregivers [13]. In combination with continuous educational methods, OMW were able to lead to an improvement in patient care [6]. In addition, two pilot evaluations [15, 19] and one evaluation study [17] with nurses from ICU proved that the staff appreciated OMW as a learning method.

Following the inspiring example of England [23], an OMW network in German-speaking countries (Austria, Germany, Luxembourg, and the German-speaking part of Switzerland) was founded in March 2018. Participation and registration in the OMW network was via email request and without any charges. The aims are free exchange, discussion and dissemination of OMW. Members of the network have access to many OMW of other settings in healthcare, which gives them the opportunity to receive additional external impulses and ideas for their own nursing activities. A newsletter with updates and new OMW are distributed within the network quarterly. Currently, about 300 people from different healthcare settings are taking part [16]. However, the benefits of this network have not been scientifically evaluated yet.

The primary objective of this evaluation study was to examine whether and to what extent the members of the OMW network used OMW for training in different settings. Secondary objectives were subjective educational gain, OMW as a training method, and OMW-related structures and processes.

## Methods

### Study design

The study is an evaluation study using a closed online survey. The survey was designed in accordance with recommendations for reporting electronical surveys ([26]; Table E1 in the online supplementary material).

### Setting

OMW network members were from different settings including ICUs, intermediate care, hospitals’ wards, long-term outpatient and inpatient care facilities and others. They are from Germany (*n* = 289), Austria (*n* = 7) and Switzerland (*n* = 5).

### Participants

All OMW network participants (*n* = 301) from German-speaking countries were invited to participate in this study. Inclusion criteria were (a) ≥ 18 years of age, (b) registered member of the OMW network. Exclusion criteria were less than 50% of the questions answered. The subjects were informed about the study and received a link to an online questionnaire, using SurveyMonkey (SurveyMonkey, San Mateo, CA, USA). The data collection was anonymous and performed in April 2020. Three reminders were sent by email.

### Variables

The primary outcome was the use of the OMW, operationalized by the questions of how many participants were using OMW, for how many years OMW have been used and how many have been created during that time.

The secondary outcome parameters are operationalized by:

- The subjective educational gain entered on a scale of 1–6 stars (6 = maximum educational gain).
- the assessment of OMW as a training method entered on a scale of 1–6 stars (6 = maximum educational gain).
- OMW-related structures and processes.

### Instrument/data

Based on previous evaluations [15, 19], we developed a questionnaire for data collection. In addition to questions about the use of OMW, sociodemographic data (country, profession, work experience etc.) were added (Table 1). The development of the questionnaire was carried out within an expert panel of nurses and researchers. The current questionnaire was standardized pretested by 13 clinicians with experience of the implementation and development of OMW for comprehensibility, linguistic adjustments and the time to fill in the questionnaire, resulting in minimal modifications.

**Table 1:** Sociodemographic data

Item^a^	Total (*n* = 191)
**Country**	***n*** **(%)**
Germany	183 (95.8)
Switzerland	5 (2.6)
Austria	3 (1.6)
**Profession**	***n*** **(%)**
Registered nurses	176 (93.6)
Physicians	3 (1.6)
Others	9 (4.8)
**Work experience**	***n*** **(%)**
0–2 years	2 (1.1)
3–4 years	3 (1.6)
5–10 years	37 (19.6)
> 10 years	147 (77.8)
**Place of work**	***n*** **(%)**
Intensive care unit	96 (50.8)
Management, research, education	46 (24.3)
General ward	15 (7.9)
Other	32 (16.9)
**Specialty**	***n*** **(%)**
Adult care	157 (84)
Pediatric care	14 (7.5)
Geriatric care	2 (1.1)
Other	14 (7.5)

^a^Data reported as *n* (%). Proportions may not add up to 100% due to rounding

### Survey

The survey was a closed online questionnaire consisting of 25 questions on 4 pages, including 151 items, with a mean of 6 items per question. The survey included closed questions with single and multiple items. In 12 questions a comment option was possible. Participants had the choice to go back and forth while answering the questions but had no access to overall results. Participants did not have to fill in every question and had an option of “not applicable” or the like. In the pretest, the mean required time to fill out the questionnaire was 8 min 30 s.

The survey assessed the following: implementation of OMW, country, profession, years of ICU experience, place of work (bedside, management, education etc.), pediatric, adult, or geriatric care, educational possibilities, number of units with OMW and discipline, number of OMW locations within one unit, OMW location, frequency of change, OMW archives, ideas for creating OMW, authorship, time since implementation, number of OMW produced, peer-review, topics, personal benefit, benefit for unit, conflicts, support, personal educational gain, overall ranking of OMW from 1 to 6 stars with 6 representing highest score.

All authors approved the final version of the survey. The survey’s items and answers in the survey were in a different order than in this publication.

### Statistical methods

The survey’s data were exported to SPSS 22.0 (IBM, Armonk, NY, USA), coded including missing data and calculated. Nominal and categorical data are reported as number and percent, ordinal data in its modus. Metrical data are reported as median and interquartile range (IQR), due to nonnormal distribution. Items were not weighted. Possible statistical relationship between the implementation of OMW and different countries, professions, years of work experience, place of work, specialty or institutions with more or less than 4 educational offers were calculated by Fisher’s exact test on the base of double-sided α = 0.05.

### Ethical considerations

No personal data were requested. All participants were informed about the voluntary, anonymous approach and the time required to answer all questions. Participation was counted as consent. In advance, the local ethics committee of the Medical Faculty of the Christian Albrechts University in Kiel approved the survey (file number D 441/20). Afterwards the study has been registered in the German register of clinical trials (DRKS00021116) on 18 March 2020 (www.drks.de).

## Results

In all, 301 members of the OMW network were invited for participation in to the study. Response rate was 62.8% (*n* = 191), completion rate 85.8% (*n* = 164). All returned questionnaires could be included.

**Fig. 2:**
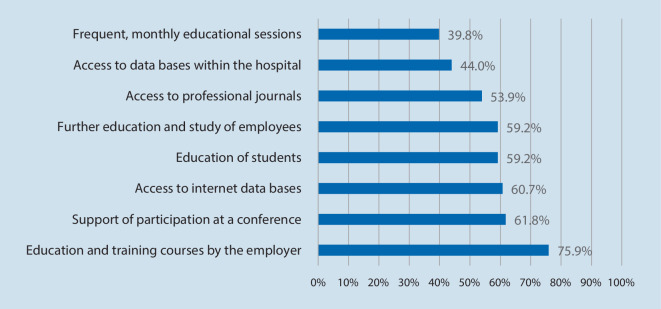
Educational trainings. Based on *n* = 191 respondents. Multiple answers. Data reported as %

Most participants were from Germany (95.8%, *n* = 183), nurses (93.6%, *n* = 176), had more than 10 years’ work experience (77.8%, *n* = 147), worked on ICU (50.8%, *n* = 96) and cared for adult patients (84%, *n* = 157; Table 1). More than half of the participants reported having educational trainings offered by the employer, education of students or supported participation at conferences (Fig. 2).

**Fig. 3:**
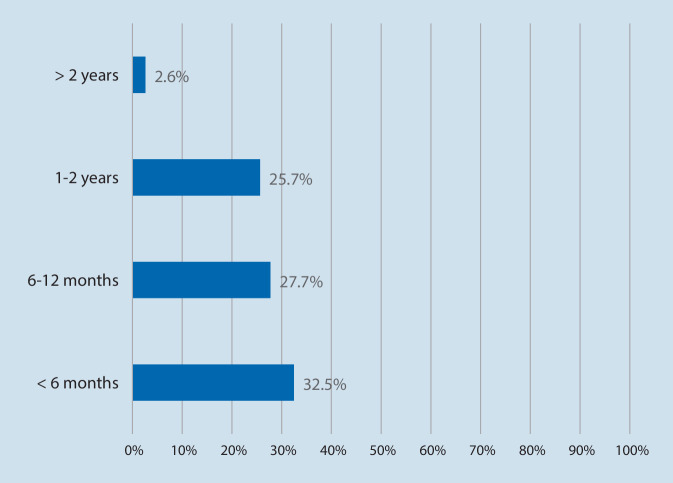
One Minute Wonder are used since. Based on *n* = 191 respondents. Multiple answers. Data reported as %. Proportions may not add up to 100% due to rounding

**Fig. 4:**
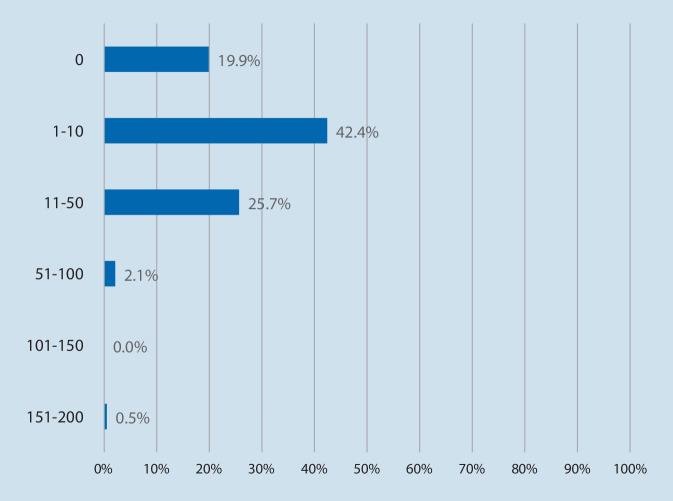
Extent of self-produced One Minute Wonder. Based on *n* = 191 respondents. Multiple answers. Data reported as *n* %. Proportions may not add up to 100% due to rounding

The majority of respondents were using OMW in their hospitals (74.2%, *n* = 141), others were planning implementation (25.8%, *n* = 49), and no one was unaffected by OMW. Related to the time OMW are used in practice, most participants reported to have been using OMW for less than 6 months (32.5%, *n* = 62) and developed 1–10 OMW (42.4%, *n* = 81; Figs. 3 and 4).

Participants ranked the median educational gain for themselves with 5 (IQR 5–6) and for their teams with 6 (IQR 5–6) of maximum 6 stars.

Participants reported benefits due to OMW. OMW extended their professional knowledge, helped to reflect their work und extended professional competence (Table 2). Some participants used OMW for writing homework or a professional thesis. Most respondents reported to have no conflicts with using OMW (56.5%, *n* = 103), while one in five participants reported that OMW led to conflicts with colleagues (19.4%, *n* = 37). These conflicts were judged as positive by the majority (19.9%, *n* = 38).

**Table 2:** Ranking of the benefits and conflicts

Item^a^ …	Total (*n* = 191)
**One Minute Wonder (OMW)**^b^ **…**	**Median (IQR)**
Extend my professional knowledge	1 (1–1)
Stimulate to reflect my work	1 (1–1.75)
Extend my professional competence	1 (1–2)
Lead to implementation of OMW	1 (1–2)
Improve quality of care in my unit/ward	1 (1–2)
Is useful for sharing evidence-based knowledge with my colleagues	1 (1–2)
Have improved my motivation	2 (1–2)
Improve cooperation in my team	2 (1–3)
Supported quality improvement projects on my unit/ward	2 (1–3)
Lead to communication with other healthcare institutions	2 (1–4)
Lead to ask for help and advice how to develop OMW	3 (2–4)
Supported quality improvement projects in my hospital	3 (2-4)
Has improved my work in a negative manner	5 (5–5)
None of the above	5 (2–5)
**Helped to write a**^c^ **…**	***n*** **(%)**
Thesis	23 (12)
Homework	21 (10.9)
Bachelor’s thesis	9 (4.7)
Dissertation thesis	9 (4.7)
Master’s thesis	4 (2)
**OMW led to conflicts …**	***n*** **(%)**
No, we have had no conflicts so far	103 (56.5)
Between my colleagues and myself	37 (19.4)
Between my knowledge and to content of OMW	21 (11)
Between different professions	13 (6.8)
Between nursing management and myself	11 (5.8)
And I rank these conflicts as very positive	38 (19.9)
And I rank these conflicts as very negative	4 (2.1)

^a^Multiple answers. Data reported as *n* (%), or median (IQR). Proportions may not add up to 100% due to rounding

^b^Options to answer were yes, rather yes, do not know, rather no, no, and coded as 1 (= yes), 2, 3, 4, and 5 (= no)

^c^Yes and rather yes from b were summarized

Questions about the structure of OMW showed that a broad range of topics, mostly nursing interventions (79.6%, *n* = 152), were covered by OMW. OMW were placed mostly on one unit/ward in the hospital (32.1%, *n* = 54), which was most often an ICU (68.1%, *n* = 130). OMW were hung up in median in 2 (IQR 1–2.7) places, most often next to the blood gas analyzer (62.3%, *n* = 119) and were stored in folders for later relocating (40.6%, *n* = 69; Table 3). A few participants reported using OMW as screensavers on monitors with frequent changes.

**Table 3:** Structure of One Minute Wonder (OMW)

Item^a^	Total (*n* = 191)
**OMW covers following subjects**	***n*** **(%)**
Nursing interventions	152 (79.6)
Diseases	136 (71.2)
Drugs	123 (64.4)
Medical interventions	98 (51.3)
Monitoring, equipment	96 (50.3)
Scales and scores	89 (46.6)
Devices	81 (42.4)
“Good to know” issues	77 (40.3)
Ethical issues	39 (20.4)
Team cooperation	35 (18.3)
Summaries of studies	33 (17.3)
Work–life balance of staff	32 (16.8)
Alternative nursing and interventions (music, pets etc.)	22 (11.5)
Family centered care	13 (6.8)
**OMW are distributed on following number of units in the hospital**	***n*** **(%)**
1	54 (32.1)
2	39 (23.2)
3	24 (14.3)
4	10 (6)
5	6 (3.6)
> 5	35 (20.8)
**OMW are distributed on following specialties**	***n*** **(%)**
Intensive care units	130 (68.1)
Intermediate care units	59 (30.9)
General ward	48 (25.1)
Anesthesia	24 (12.6)
Emergency department	13 (6.8)
Research and education	12 (6.3)
Management	11 (5.8)
Living area	5 (2.6)
Other	14 (7.3)
**Places of OMW**	***n*** **(%)**
Blood gas analyzer	119 (62.3)
Centre of unit	63 (33)
Staff pantry	61 (31.9)
Kitchen	33 (17.3)
Storage	32 (16.8)
Staff’s washroom	22 (11.5)
Drinks trailer	4 (2.1)
Other	35 (18.3)
**Archives of previous OMW**	***n*** **(%)**
OMW are stored in a folder	69 (40.6)
OMW are stored electronically	38 (22.4)
OMW can be read	23 (13.5)
OMW are stored in different medias	8 (4.7)
OMW are not stored	31 (18.2)

*OMW* One Minute Wonder

^a^Multiple answers. Data reported as *n* (%). Proportions may not add up to 100% due to rounding

Participants reported OMW-related processes. OMW were most often infrequently changed (43.5%, *n* = 74). Attendees reported receiving their inspiration for developing OMW most often from the OMW network (63.9%, *n* = 122). Creators of OMW were most often nurses of the unit/ward (53.9%, *n* = 103). OMW were reviewed for quality before publishing by the leading nurses (42.9%, *n* = 82). Developing OMW was most often honored by the acknowledgement of overtime (28.8%, *n* = 55). Some participants also reported that development was done in their regular working time (Table 4).

**Table 4:** Processes related to One Minute Wonder (OMW)

Item^a^	Total (*n* = 191)
**Frequency of renewing OMW**	***n*** **(%)**
Infrequently	74 (43.5)
Once per 1 week	9 (5.3)
Once per 2 weeks	52 (30.6)
Once per 4 weeks	20 (10.5)
Other	15 (7.8)
**Inspiration for development of OMW is generated by**	***n*** **(%)**
OMW network	122 (63.9)
Questions by team, meetings	99 (51.8)
Journals	75 (39.3)
Conferences	55 (28.8)
Further education, study	50 (26.2)
Team leaders	28 (14.7)
Social media	16 (8.4)
Other	25 (13.1)
**Creators of OMW**	***n*** **(%)**
Nurses of the unit/ward	103 (53.9)
OMW delegate	51 (26.7)
Staff of quality management	18 (9.4)
Internal education/internal training	14 (7.3)
Students	13 (6.8)
Other professions	13 (6.8)
Trainees	9 (4.7)
Other	48 (25.1)
**OMW are reviewed before publishing by**	***n*** **(%)**
Leading nurse	82 (42.9)
Specific experts	63 (33)
Themselves (respondent of survey)	63 (33)
Leading physician	37 (19.4)
OMW project group	36 (18.8)
OMW are not reviewed	12 (6.3)
Other	12 (6.3)
**The creation and development of OMW is honored by**	***n*** **(%)**
Acknowledgement of overtime	55 (28.8)
Support in literature search	18 (9.4)
Exemption from work	14 (7.3)
Financial bonus	2 (1)
A certification	1 (0.5)
Other	23 (12)

^a^Multiple answers. Data reported as *n* (%). Proportions may not add up to 100% due to rounding. In some items, data were missing, all < 5%

There were no significant differences for the implementation of OMW and different countries (*p* = 0.255), professions (*p* = 0.421), years of work experience (*p* = 0.831), place of work (*p* = 0.224), specialty (0.222), or institutions with more or less than 4 educational offers (*p* = 0.620).

## Discussion

The electronical survey within the German-speaking OMW network including nearly 200 respondents showed that three quarter of clinicians were using OMW in practice, while one quarter is planning implementation. Regarding the time OMW have been used in practice, most participants reported having used OMW for less than 6 months and developed fewer than 10 OMW. OMW were hung up next to the blood gas analyzer or used as screen savers on monitors. OMW topics were most often nursing interventions, diseases, drugs, and others. Authors of OMW were most often nurses who were inspired by the OMW network or by questions of the team. Participants reported that OMW extended their professional knowledge, stimulated them to reflect on their work, and were highly ranked for sharing best practice knowledge.

The results show that three quarters of the participated clinicians used OMW most frequently in hospital settings. Considering that the original idea of the OMW also originates from the hospital setting and has been significantly communicated in this environment in the German-speaking region [8, 19], this result is not surprising. The usage mainly takes place in ICU. This is probably due to the fact that the network was founded by ICU staff and was therefore also presented to this group, for example at conferences and by publications [12, 15]. The educational offers in the hospitals indicate the extent of support of their employees (Fig. 2). At the same time, the results show that other healthcare facilities, such as emergency services, also use OMW to impart knowledge (6.8%, *n* = 13) and these facilities also communicate this through publications [8]. The work of Herrmann et al. [12] shows that the method has also established itself in other specialist areas, for example in a neonatal ICU. Anecdotally, we notice an increasing interest for OMW in other settings such as nursing facilities, ambulant care, or hospice care. Nevertheless, the reasons for nonparticipation in the OMW network can be manifold such as use of other educational methods, no knowledge about the network, no interest, language barriers, time und staff constraints, and others.

The number of members of the OMW network shows that the method is frequently used in German-speaking countries and represents an effective method of knowledge transfer. Given that the network has only existed since March 2018, the high number of respondents intending to implement this method is consistent. The fact that the OMW has been used by many for less than 6 months is also attributable to the age of the network that continues to grow. Since this study in April 2020, more than 80 new members were registered in the OMW network as of March 2021. A further interpretation of the high number of interested parties is that this method is highly accepted in practice. At the time of the survey, the majority of respondents had created 1–10 OMW. This depicts the high willingness of nursing staff to actively participate in this educational method. A further indication of the high acceptance of this low-threshold method is also shown by the fact that this approach is also be used in times of crisis [14] and is supported in this context by the German Interdisciplinary Association for Intensive Care and Rescue Medicine and further professional associations in nursing and medicine [11]. The website of the network (https://omw.hdz-nrw.de) currently offers learning posters on the topic of COVID-19 free of charge.

Overall, the personal educational gain through the OMW is rated by the respondents with a median of 5 out of 6 stars. This shows that this method certainly has a self-reported effect for the participants and is frequently used in practice. On the whole, the OMW is given a median of 6 out of 6 stars for the benefit to the team. It is interesting to note that the OMW is also used for critical reflection of one’s own work and leads to constructive discussions within the team, which in itself has its own learning effect [1]. Since the critical reflection of external evidence in relation to the individual patient arrangement and thus to one’s own work is a cornerstone of evidence-based care [3] and WBL [1, 22]. Billett pointed out that there are different activities during the regular work “… that are potentially pedagogically rich” [4]. OMW seems to be that for the participants in this study. Thus, the OMW is not only an educational tool, but also an instrument for quality-oriented practice development.

Regarding educational offers, effectiveness always depends on the scope, the didactic preparation, the target groups orientation and the general conditions in the context of the setting [23]. As OMW always present a condensed presentation of complex topics, the daily working conditions have to be considered when creating OMW [10]. A positive attitude towards education and individual training measures also supports the absorption and implementation of new knowledge [10]. Krüger et al. [17] have shown their study that 73.55% of those questioned confirmed the contents of OMW as helpful in their daily work. Lehnen et al. [19] could point out that 80% of the respondents remember the contents of OMW. Due to the small sample size of the first two cross-sectional surveys of Krüger [15] (*n* = 43) and Lehnen et al. [19] (*n* = 55) and the evaluation study of Krüger et al. [17] (*n* = 189), the external validity is limited.

### Strengths and limitations

A strength of this survey is the high response rate of participants. Another strength is the widespread OMW network, covering a broad range implementation in different types of ICUs. Our study has some limitations. Since our results are limited to answers of participants from the OMW network, respondents possibly represent a recruitment and/or performance bias. However, the survey was anonymous and participants had no benefit for providing best answers. Second, we could not prevent participants from multiple entries, but due to the noneconomical or nonpolitical nature of this survey, this is unlikely to have happened. The report of this survey is limited to German-speaking population and culture; in countries with English as the first language, or different medical education, the perception of such an educational method may be different. Another limit is the self-constructed questionnaire without any data about its validity, reliability or objectivity. However, the questionnaire has been developed within an expert panel and was standardized pretested by 13 clinicians as an important step for practical use.

## Conclusions for practice

- The majority of the survey’s respondents implemented OMW in their units and shared developed OMW within the network. The sharing process is free and all participants can have a benefit. Furthermore, this method is feasible, simple and helped clinicians to extend their professional knowledge.
- OMW can cover a broad range of topics, should be changed frequently and stored in a folder for relocating later.
- OMW support professional reflection, continuous education of the multiprofessional team as well as the implementation of new procedures and thus put knowledge into practice.
- Different settings in critical care should use OMW in practice.

## Supplementary Information

Table E1: Criteria for reporting electronical surveys

## Abbreviations

OMW: One Minute Wonder
WBL: Work-based learning

## Declarations

For this article all investigations on humans described were carried out with the approval of the responsible ethics committee, in accordance with national law and the declaration of Helsinki in 1975 (in the current, revised version). Consent for publication: The authors affirm that the participant provided informed consent for publication of the image in Fig. 1.
