# Supplementary material for: Learning in one minute: survey of the One Minute Wonder Network
Source: Med Klin Intensivmed Notfmed. 2022 Jan 4;117(2):159–67. doi: 10.1007/s00063-021-00892-3 (PMC8725639; doi:10.1007/s00063-021-00892-3)
Supplement: Supplementary file 1 — Table E1: Criteria for reporting electronical surveys [file 63_2021_892_MOESM1_ESM.pdf]

Supplementary Information

Title
Learning in one minute: Survey of the One Minute Wonder Network

Journal Name
Medizinische Klinik - Intensivmedizin und Notfallmedizin

Authors

Lars Krüger M.Sc.[1*], Thomas Mannebach[2], Marianne Rahner M.Sc.[3], Fabian Timpe M.Sc.[4], Franziska Wefer M.Sc.[5], Dr. rer.hum.biol. Peter Nydahl[6]

1. Continuing education intensive care, surgical intensive care unit E 0.1, Heart and Diabetes Center NRW, University Hospital of the Ruhr University Bochum, Georgstraße 11, 32345 Bad Oeynhausen, Germany. lkrueger@hdz-nrw.de https://orcid.org/0000-0003-4234-0022
2. Surgical intensive care unit E 0.1, Heart and Diabetes Center NRW, University Hospital of the Ruhr University Bochum, Georgstraße 11, 32345 Bad Oeynhausen, Germany. tmannebach@hdz-nrw.de https://orcid.org/0000-0001-9217-3299
3. Institute for Public Health and Nursing Research, University Bremen, Grazer Straße 4, 28359 Bremen, Germany. rahner@uni-bremen.de https://orcid.org/0000-0003-1096-2644
4. Care development, care directorate, St. Bernward hospital GmbH, Hildesheim, Treibestraße 9, 31134 Hildesheim, Germany. f.timpe@bernward-khs.de
5. Care development, care directorate, Heart and Diabetes Center NRW, University Hospital of the Ruhr University Bochum, Georgstraße 11, 32345 Bad Oeynhausen, Germany. fwefer@hdz-nrw.de
6. Nursing research; department of anaesthesia and operative intensive care medicine, University Hospital Schleswig-Holstein, House V40, Brunswiker Str. 10, 24105 Kiel, Germany. Peter.Nydahl@uksh.de https://orcid.org/0000-0002-5178-0364

***Corresponding author**
Lars Krüger, M.Sc.
Continuing education intensive care, surgical intensive care unit E 0.1
Heart and Diabetes Center NRW, University Hospital of the Ruhr University Bochum,
Georgstraße 11, 32345 Bad Oeynhausen, Germany
E-Mail: lkrueger@hdz-nrw.de https://orcid.org/0000-0003-4234-0022
Phone: +49 5731/973280

Content
Table E1: Criteria for reporting electronical surveys

Table E1: Criteria for reporting electronical surveys [1]

Checklist Item	Explanation	Page[1]
Title and abstract		
Design of the study stated	Are the words 'questionnaire' or 'survey' stated in the title and/or abstract?	Titlepage
Introduction		
Background provided	Did the authors present a well-written background to their research?	7-8
Purpose/aim of paper explicitly stated	Did the authors identify a specific purpose, aim, goal, or objective of the study?	8
Tool of measurement		
Description of the questionnaire	Did they provide access to the questionnaire items used in the study in either the article, appendices, or an online supplement?	8-10
Existing tool	Existing tool = (the questionnaire was constructed by others and the authors just used it).	9-10
Psychometric properties presented	Psychometric properties = (were the reliability and validity of the existing questionnaire mentioned?)	n/a
Existing tool, references to original work provided	Did they provide a reference to the existing tool (questionnaire) that they used?	9
New tool, procedures to develop and pre-test provided	Did studies that developed a novel questionnaire clearly describe the development process and/or describe the methods used to pre-test the tool? Have the usability and technical functionality of electronic questionnaires been tested before fielding them?	8-10
New tool, reliability and validity reported	Did they report the reliability and validity of the developed questionnaire?	n/a
Description of the scoring procedures provided	Did papers which used survey instruments that required scoring provide a description of the scoring procedures?	9-10
Recruitment process and sample description		
Description of survey population and sample frame	Survey population = the main target of the study. Sample frame = the methods they used to reach this target. For instance, if a study wanted to detect the prevalence of hypertension in elderly people above 65 years old, and researchers went to 10 elderly care centers and randomly recruited 50 old men from the registry of each of those centers. This means that our survey population is elderly people above 65, and the sample frame is the 10 care centers registry.	8-10

Description of representativeness of the sample	Is a description of whether the sample will represent the whole population provided?	n/a
Sample size calculation or rationale/justification presented	Did they mention a description of their sample size calculation, such as providing a formula or a rationale?	n/a
Open survey versus closed survey*	An “open survey” is a survey that is available for each visitor of a site, while a closed survey is only open to a sample which the investigator knows (password-protected surveys and surveys in which participants are invited by individual survey links).	9-10
Advertising the survey	How/where was the survey announced or advertised? It is important to know the wording of the announcement as it will heavily influence who chooses to participate. Ideally, the survey announcement should be published as an appendix.	8-10
Incentives	Were any incentives offered (e.g., monetary, prizes, or non-monetary incentives such as an offer to provide the survey results)?	n/a
**Survey Administration**		
Mode of administration	Did the authors specify how they administered the survey to the participant?	8-10
Information on the type and number of contacts provided	This refers to the initial contact between the participants and the researchers; type (i.e. phone, e-mail, postal mail, online/offline advertisement), number of contacts (i.e. how many times they tried to contact them?)	8-9
Description of who approached potential participants	Description of who was identified as the organization/group urging potential research subjects for their participation in the survey. Did they mention the group giving money for participants to enter the study or the group who convinced them to participate and explained the study to them?	8-9
Context*	Description of the Web site (for mailing list/newsgroup) on which the survey was posted. What is the Web site about, who is visiting it, what are visitors normally looking for? Discuss to what degree the content of the Web site could pre-select the sample or influence the results. For example, a survey about vaccination on an anti-immunization Website will have different results from a Web survey conducted on a government Website.	8-10
Preventing multiple entries from the same individual*	This is mostly concerned with open surveys. Did they indicate whether cookies were used to assign a unique user identifier to each client's computer? If so, did they mention the page on which the cookie was set and read, and for how long was the cookie valid? Were duplicate entries avoided by preventing users' access to the survey twice? or were duplicate database entries having the same user ID eliminated before analysis? In the latter case, which entries were kept for analysis (eg, the first entry or the most recent)?<br>Did they indicate whether the IP address of the client's computer was used to identify potential duplicate entries from the same user? If so, did they mention the period of time for which no two entries from the same IP address were allowed (e.g., 24 hours)? Were duplicate entries avoided by preventing users with the same IP address access to the survey twice; or were duplicate database entries having the same IP address within a given period of time eliminated before analysis? If the latter, which entries were kept for analysis (eg, the first entry or the most recent)?	11-15

	Did they indicate whether other techniques to analyze the log file for identification of multiple entries were used? In “closed” (non-open) surveys, users need to login first and it is easier to prevent duplicate entries from the same user. Did they describe how this was done? For example, was the survey never displayed a second time once the user had filled it in, or was the username stored together with the survey results and later eliminated? If the latter, which entries were kept for analysis (eg, the first entry or the most recent)?	
Mandatory/voluntary	Was it a mandatory survey to be filled in by every visitor who wanted to enter the Website, or was it a voluntary survey?	n/a
Time/Date	In what timeframe were the data collected?	8-9
Randomization of items or questionnaires	To prevent biases items can be randomized or alternated.	11-15
Completeness check	It is technically possible to do consistency or completeness checks before the questionnaire is submitted. Was this done? If yes, how (usually JAVAScript)? An alternative is to check for completeness after the questionnaire has been submitted (and highlight mandatory items). If this has been done, it should be reported. All items should provide a non-response option such as “not applicable” or “rather not say”, and selection of one response option should be enforced.	9-10
Review step*	Did they state whether respondents were able to review and change their answers? (e.g., through a "Back" button or a "Review step" which displays a summary of the responses and asks the respondents if they are correct).	9-10
Response rates		
Response rate reported	For web-based surveys, authors should count the unique number of visitors who visit the first page of the survey (or the informed consents page, if present) divided by the number of people who filled in the first survey page (or agreed to participate). This can also be called “recruitment” rate.	n/a
Completion rate (Ratio agreed to participate/finished survey)	The number of people agreeing to participate (or submitting the first survey page) divided by the number of people submitting the last questionnaire page. This is only relevant if there is a separate “informed consent” page or if the survey goes over several pages. This is a measure for attrition. Note that “completion” can involve leaving questionnaire items blank. This is not a measure for how completely questionnaires were filled in. (If you need a measure for this, use the word “completeness rate”.)	9-11
Unique site visitor*	If view rates or participation rates are provided, authors need to define how you determined a unique visitor. There are different techniques available, based on IP addresses or cookies or both.	n/a
Adaptive questioning	Did they use adaptive questioning (certain items are only conditionally displayed based on responses to other items) to reduce number and complexity of the questions. Can affect the response rate.	n/a
Number of Items	What was the number of questionnaire items per page? The number of items is an important factor for the completion rate.	9-10
Analysis		

Methods of data analysis	Was a description of the variables that were analyzed, how they were manipulated, and the statistical methods that were used provided?	9-10
Method for analysis of nonresponse error provided	_	4-6
Definitions for complete versus partial completions provided	Was a definition or cut-off limit for partial completion of questionnaires provided?	n/a
Methods for handling item missing data provided	Were the methods for handling item missing data provided?	8-10
Questionnaires submitted with an atypical timestamp	Some investigators may measure the time people needed to fill in a questionnaire and exclude questionnaires that were submitted too soon. Did they specify the timeframe that was used as a cut-off point, and describe how this point was determined?	n/a
Statistical correction	Did they indicate whether any methods such as weighting of items or propensity scores have been used to adjust for the non-representative sample?	10
Results		
All respondents accounted for	Did they report the sample disposition (i.e., describing the number of complete and partial returned questionnaires according to the number of potential participants known to be eligible, of unknown eligibility, or known to be ineligible)?	10
Information on how non-respondents differ from respondents provided	_	n/a
Results clearly presented	_	11-12
Results address objectives	_	11-12
Discussion		
Results summarized referencing study objectives	_	13
Strengths of the study stated	_	15
Limitations of the study stated	_	15
Generalizability of results discussed	Did they include any discussion on the generalizability of their results?	15
**Ethical Quality Indicators**		
Study funding reported		Titlepage

Research Ethics Board (REB) review reported		11
Subject consent procedures reported		Titlepage
Data protection*	If any personal information was collected or stored, did they describe what mechanisms were used to protect unauthorized access?	n/a

Abbreviations: n/a not applicable

The (*) symbol refer to items that are more concerned with web-based studies.

[1] The page numbers refer to the manuscript version post-peer review, but prior to copy-editing and typesetting.

Reference

1. Turk T, Elhady MT, Rashed S et al. (2018) Quality of reporting web-based and non-web-based survey studies: What authors, reviewers and consumers should consider. PLoS One 13:e0194239
